# Membrane plasmalogen composition and cellular cholesterol regulation: a structure activity study

**DOI:** 10.1186/1476-511X-9-62

**Published:** 2010-06-14

**Authors:** Rishikesh Mankidy, Pearson WK Ahiahonu, Hong Ma, Dushmanthi Jayasinghe, Shawn A Ritchie, Mohamed A Khan, Khine K Su-Myat, Paul L Wood, Dayan B Goodenowe

**Affiliations:** 1 Phenomenome Discoveries Inc. and Phreedom Pharma, 204-407 Downey Road, Saskatoon, SK, S7N 4L8, Canada

## Abstract

**Background:**

Disrupted cholesterol regulation leading to increased circulating and membrane cholesterol levels is implicated in many age-related chronic diseases such as cardiovascular disease (CVD), Alzheimer's disease (AD), and cancer. *In vitro *and *ex vivo *cellular plasmalogen deficiency models have been shown to exhibit impaired intra- and extra-cellular processing of cholesterol. Furthermore, depleted brain plasmalogens have been implicated in AD and serum plasmalogen deficiencies have been linked to AD, CVD, and cancer.

**Results:**

Using plasmalogen deficient (NRel-4) and plasmalogen sufficient (HEK293) cells we investigated the effect of species-dependent plasmalogen restoration/augmentation on membrane cholesterol processing. The results of these studies indicate that the esterification of cholesterol is dependent upon the amount of polyunsaturated fatty acid (PUFA)-containing ethanolamine plasmalogen (PlsEtn) present in the membrane. We further elucidate that the concentration-dependent increase in esterified cholesterol observed with PUFA-PlsEtn was due to a concentration-dependent increase in sterol-O-acyltransferase-1 (SOAT1) levels, an observation not reproduced by 3-hydroxy-3-methyl-glutaryl-CoA (HMG-CoA) reductase inhibition.

**Conclusion:**

The present study describes a novel mechanism of cholesterol regulation that is consistent with clinical and epidemiological studies of cholesterol, aging and disease. Specifically, the present study describes how selective membrane PUFA-PlsEtn enhancement can be achieved using 1-alkyl-2-PUFA glycerols and through this action reduce levels of total and free cholesterol in cells.

## Background

A breakdown in cholesterol homeostasis has adverse effects at the cellular level, as well as in the context of the organism. Altered cholesterol content in cells affects membrane fluidity, which has drastic effects on cellular function, signal transduction, and intercellular communication events [1,2]. Elevated levels of circulating cholesterol have been linked with the formation of atherosclerotic plaques, and is a risk factor for cerebrovascular lesions and coronary heart disease [3,4]. Apolipoprotein E4 (ApoE4), a vehicle for cholesterol transport, is a major risk factor for sporadic Alzheimer's disease (AD), demonstrating a link between cholesterol and cognition [5]. Increase in cholesterol in tumor tissue is a common underlying feature in a number of cancers; safety data from randomized clinical trials of cholesterol lowering statins demonstrated lower incidences of melanoma, colorectal, breast and prostate cancers, reviewed by Hager and coworkers [6].

Cholesterol exists in two mutually exclusive pools in the body separated by the blood brain barrier. Within each pool it can be found either in a free (unesterified) state, or it can exist as esters. Brain cholesterol is synthesized *de novo*, and accounts for 25% of the total body cholesterol, wherein it exists primarily as free cholesterol in myelin and the plasma membranes of glial cells and neurons [7,8]. The remaining cholesterol is accounted for in tissues and in circulation. The plasma membrane of cells is predominantly composed of unesterified cholesterol, which is enriched in microdomains called lipid rafts, key structural requirements for signal transduction. Circulating cholesterol on the other hand is coupled with lipoproteins (chylomicrons, VLDL, LDL and HDL). Chylomicrons, VLDL and LDL serve as vehicles for the movement of dietary cholesterol to the liver for removal from circulation. HDL, synthesized by the liver and intestine, is the vehicle for the transport of tissue cholesterol to the liver for excretion, a process **c**alled reverse cholesterol transport (reviewed by Martins and coworkers) [9].

Plasmalogens are a class of glycerophospholipids characterized by a vinyl-ether linkage at the sn-1 position and an acyl linkage at the sn-2 position of the glycerol backbone. Besides contributing to membrane structural integrity, plasmalogens are involved in multiple cellular functions such as vesicle formation and membrane fusion [10-12], ion transport [13-15] and generation of secondary signal mediators such as platelet activating factor (PAF) [16,17]. Presence of the vinyl ether bond imparts antioxidant properties to these molecules which mitigates free radical based cellular damage [18-21].

The multitude of functions attributed to this class of molecules implicates it in a number of human disorders ranging from peroxisomal disorders such as Zellwegger syndrome, rhizomelic chondrodysplasia punctata (RCDP), infantile Refsum disease and cholesterol storage disorders such as Neiman-Pick type C disease to Down's syndrome and Alzheimer's disease [22-28]; Ethanolamine plasmalogen depletion has been observed in post-mortem brains of AD subjects [29,30] and in the serum of subjects suffering from AD [31], cardiovascular disease [32], and cancer [33]

Studies have shown that brain and circulating plasmalogens negatively correlate with age [34-36]. Additionally, plasmalogens have been linked with altered cholesterol processing [37-39]; a plasmalogen-deficient cell exhibits lower esterified cholesterol and a lower rate of HDL-mediated cholesterol efflux. Meaba and coworkers recently showed a link between plasmalogens and Apo A1 and A2, the major components of HDL [35].

These observations prompted us to investigate the relationship between membrane plasmalogen level and cholesterol regulation using both plasmalogen deficient (NRel-4) and sufficient (HEK293) cell lines. A novel species-specific plasmalogen restorative/augmentation approach was applied to both cell types and the resulting effect on cholesterol (total, esterified, and free) and sterol-O-acyltransferase-1 (SOAT1 encodes acyl-coenzyme A:cholesterol acyl transferase, ACAT, a critical membrane bound cholesterol processing enzyme), levels ascertained. This report identifies the use of plasmalogens in achieving cholesterol homeostasis as an alternative to statin therapy.

## Materials and Methods

### Syntheses of Compounds for Structure Activity Relationship Study

**Figure 1:**
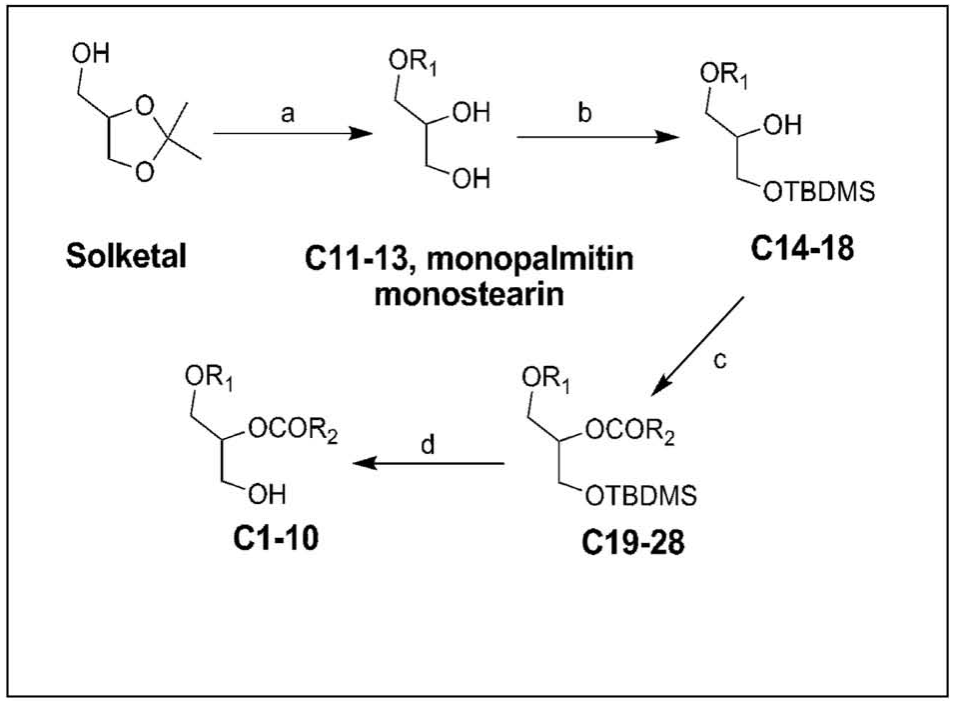
**Scheme showing the syntheses of phosphoethanolamine plasmalogen precursors C1-3, C6-10, and diacylglycerols C4 and C5, test compounds for the study**. Reagents: (a) i R_1_Br, NaH/DMF or R_1_SO_4_CH_3_, NaH/THF, reflux; (ii) 10% HCl, reflux; (b) TBDMS-Cl, imidazole/DMF; (c) R_2_COCl, DMAP, Pyridine, Toluene; (d) TBAF/THF, Imidazole, - 20°C

The compounds used for this structure activity relationship study were synthesized from readily available starting materials as shown in the synthetic scheme (Figure 1) and in Table 1.

**Table 1:** List of compounds synthesized for the SAR study.

	Compound	***sn*-1 (R**_**1**_**)**	***sn*-2 (R**_**2**_**)**	*sn*-3
**1**	cis-(±)-2-*O*-docosahexaenoyl-1-*O*-hexadecylglycerol	**16:0 (alkyl)**	**DHA**	**OH**

**2**	cis-(±)-2-*O*-docosahexaenoyl-1-*O*-octadecylglycerol	**18:0 (alkyl)**	**DHA**	**OH**

**3**	cis-(±)-2-*O*-docosahexaenoyl-1-*O*-octadec-9-enylglycerol	**18:1 (alkyl)**	**DHA**	**OH**

**4**	cis-(±)-2-*O*-docosahexaenoyl-1-*O*-palmitoylglycerol	**16:0 (acyl)**	**DHA**	**OH**

**5**	cis-(±)-2-*O*-docosahexaenoyl-1-*O*-stearoylglycerol	**18:0 (acyl)**	**DHA**	**OH**

**6**	cis-(±)-2-*O*-stearoyl-1-*O*-hexadecylglycerol	**16:0 (alkyl)**	**18:0**	**OH**

**7**	cis-(±)-2-*O*-oleoyl-1-*O*-hexadecylglycerol	**16:0 (alkyl)**	**18:1**	**OH**

**8**	cis-(±)-2-*O*-linoleoyl-1-*O*-hexadecylglycerol	**16:0 (alkyl)**	**18:2**	**OH**

**9**	cis-(±)-2-*O*-α-linolenoyl-1-*O*-hexadecylglycerol	**16:0 (alkyl)**	**18:3**	**OH**

**10**	cis-(±)-2-*O*-arachidonoyl-1-*O*-hexadecylglycerol	**16:0 (alkyl)**	**20:4**	**OH**

All molecules had a glycerol backbone, and differed in the *sn*-1 and *sn*-2 fatty acid acids. The *sn*-1 fatty acid was linked to glycerol either by an alkyl or acyl linkage.

### General Methods

All chemicals and solvents were purchased from Sigma-Aldrich Canada Ltd., Oakville, ON., VWR Canada and Nu-Chek Prep., Elysian, MN. All solvents used were anhydrous. Analytical thin layer chromatography (TLC) was carried out on precoated silica gel TLC aluminum sheets (EM science, Kieselgel 60 F_254_, 5 × 2 cm × 0.2 mm). Compounds were visualized under UV light (254/366 nm) or placed in iodine vapor tank and by dipping the plates in a 5% aqueous (w/v) phosphomolybdic acid solution containing 1% (w/v) ceric sulfate and 4% (v/v) H_2_SO_4_, followed by heating. Flash column chromatography was carried out using silica gel, Merck grade 60, mesh size 230-400, 60 Å. NMR spectra were recorded on Bruker Avance spectrometers; for ^1^H (500 MHz), δ values were referenced to CDCl_3 _(CHCl_3 _at 7.24 ppm) and for ^13^C NMR (125.8 MHz) referenced to CDCl_3 _(77.23 ppm). Coupling constants (*J*) are reported to the nearest 0.5 Hz. High resolution mass spectral data were obtained on Bruker Apex 7T Fourier transform ion cyclotron resonance mass spectrometer (FT-ICRMS) with atmospheric pressure chemical ionization in the positive mode (HRAPCI-MS). MS/MS data collected using QStar XL TOF mass spectrometer with atmospheric pressure chemical ionization (APCI) source in the positive mode and collision energy of 20 and 35 V. Fourier transform infra-red (FTIR) spectra were recorded on Bio-Rad FTS-40 spectrometer using the diffuse reflectance method on samples dispersed in KBr. Refer synthetic scheme (Figure 1) and Table 1 for details of compounds synthesized.

### General procedure for synthesis of C11-13

To sodium hydride (1.85 g, 60% dispersed in mineral oil) under argon was added anhydrous *N, N-*dimethylformamide (DMF, 10 ml) at room temperature (RT). Solketal (16.7 mmol) in 10 ml anhydrous DMF was then added dropwise with constant stirring. 1-Bromohexadecane, 1-bromooctadecane or octadec-9-enylmethane sulphonate (16.7 mmol) [40,41] dissolved in anhydrous DMF (10 ml) was then added to the reaction mixture dropwise and stirred for 48 hours. The reaction mixture was poured into cold ice water (100 ml) and extracted with hexane (100 ml, 3×). After drying over anhydrous Na_2_SO_4 _and removal of solvent, the crude product was treated with 10% HCl solution (40 ml) and refluxed at 120°C for 30 min. The reaction mixture was then kept at RT for 24 hours. The off white lumps and the mother liquor was extracted with diethylether (100 ml, 3×) washed successively with saturated aqueous NaHCO_3 _(100 ml) and water (100 ml), dried over anhydrous Na_2_SO_4 _and the solvent removed under reduced pressure to obtain the products **C11-13**. Satisfactory spectral and analytical data were obtained [42].

### General procedure for synthesis of C14-18

Each of compounds **C11-13**, monopalmitin and monostearin (1.63 mmol) was dissolved separately in anhydrous DMF (2.00 ml) followed by the addition of imidazole (3.72 mmol) and *tert*-butyl dimethylsilyl chloride (1.88 mmol). The reaction mixture was stirred at RT for 24 hours, poured into water (100 ml) and extracted with diethyl ether (100 ml, 3×). After removal of solvent, the crude product was chromatographed on silica gel using CH_2_Cl_2_-MeOH to obtain the products [43].

### General procedure for synthesis of C19-28

To a mixture of each of **C14-18 **(0.578 mmol), anhydrous pyridine (0.35 ml), catalytic amount of dimethylaminopyridine (0.100 mmol) and toluene (5 ml) was added the appropriate acyl chloride like (4*Z*,7*Z*,10*Z*,13*Z*,16*Z*,19*Z*)-docosa-4,7,10,13,16,19-hexaenoyl chloride (0.576 mmol) dropwise under argon and stirred at RT for 48 hours. The reaction mixture was poured into water (100 ml), extracted with diethyl ether (100 ml, 3×), washed successively with 0.25 M H_2_SO_4 _solution (100 ml), saturated aqueous NaHCO_3 _(100 ml) and water (100 ml). After drying over anhydrous Na_2_SO_4 _and removal of solvent, the crude product was subjected to chromatography on silica gel using hexane-CH_2_Cl_2 _mixtures to obtain the products.

### General procedure for synthesis of C1-10

To a mixture of each of **C19-28 **(0.272 mmol) and THF (2 ml) was added imidazole (0.942 mmol) and 0.80 ml of 1.0 M TBAF in THF dropwise at - 20°C and kept at this temperature for 24 hours with constant stirring. The reaction mixture was then passed through a plug of silica gel and eluted with cold diethylether (10 ml, - 20°C). After removal of solvent, the crude products were chromatographed on silica gel using hexane - ethyl acetate mixtures to obtain the products [44,45].

#### Spectral data for cis-(±)-2-*O*-Docosahexaenoyl-1-*O*-hexadecylglycerol (C1)

Obtained from **C19**, as light yellow oil; 144.6 mg (67%); TLC: *R*_f _= 0.65 (CH_2_Cl_2_:MeOH, 95:5 v/v); ^1^H NMR (CDCl_3_): δ in ppm 0.86 (3H, t, *J *= 7.0 Hz), 0.95 (3H, t, *J *= 7.0 Hz), 1.23-1.27 (24H, m), 1.52-1.56 (4H, m), 2.06 (2H, m), 2.17 (1H, br s), 2.38-2.41 (4H, m), 2.79-2.82 (10H, m), 3.38-3.47 (2H, m), 3.56-3.63 (2H, m), 3.76-3.79 (2H, m), 4.98 (1H, quintet, *J *= 5.0 Hz), 5.33-5.38 (12H, m); ^13^C NMR (CDCl_3_): δ in ppm 14.3, 14.5, 20.8, 22.9, 23.0, 25.8, 25.9, 26.3, 29.6, 29.7, 29.8 (3), 29.9, 32.1, 34.5, 63.2, 70.1, 72.1, 73.3, 127.2, 128.0, 128.1, 128.2, 128.3 (2), 128.5 (3), 128.8, 129.6, 132.3, 173.1; FT-IR (cm^-1^) 3401 (br), 2931, 2850, 1728; HRAPCI-MS *m/z*: measured 627.5353 ([M + H]^+^, calcd. 627.5352 for C_41_H_71_O_4_); APCI-MS/MS *m/z*: 627 ([M + H]^+^, 80%), 609 (20%), 385 (40%), 329 (15%), 311 (100%), 293 (90%), 269 (35%), 75 (70%).

#### Spectral data for cis-(±)-2-*O*-Docosahexaenoyl-1-*O*-octadecylglycerol (C2)

Obtained from **C20**, as light yellow oil; 75.2 mg (66.3%); TLC: *R*_f _= 0.80 (CH_2_Cl_2_:MeOH, 95:5 v/v); ^1^H NMR (CDCl_3_): δ in ppm 0.86 (3H, t, *J *= 7.0 Hz), 0.95 (3H, t, *J *= 7.0 Hz), 1.23-1.27 (28H, m), 1.50-1.56 (4H, m), 2.05 (2H, m), 2.19 (1H, br s), 2.37-2.42 (4H, m), 2.78-2.83 (10H, m), 3.38-3.46 (2H, m), 3.55-3.63 (2H, m), 3.76-3.79 (2H, m), 4.98 (1H, quintet, *J *= 5.0 Hz), 5.33-5.38 (12H, m); ^13^C NMR (CDCl_3_): δ in ppm 14.3, 14.5, 20.8, 22.9, 23.0, 25.8 (2), 25.9, 26.3, 29.6, 29.7, 29.8 (3), 29.9, 32.1, 34.5, 63.2, 70.1, 72.1, 73.3, 127.2, 128.0, 128.1, 128.2, 128.3 (2), 128.5 (3), 128.8, 129.6, 132.3, 173.1; FT-IR (cm^-1^) 3397 (br), 2954, 2861, 1738.; HRAPCI-MS *m/z*: measured 655.5665 ([M + H]^+^, calcd. 655.5665 for C_43_H_75_O_4_); APCI-MS/MS *m/z*: 655 ([M + H]^+ ^75%), 637 (30%), 385 (40%), 329 (15%), 311 (100%), 293 (60%), 269 (20%), 75 (60%).

#### Spectral data for cis-(±)-2-*O*-Docosahexaenoyl-1-*O*-octadec-9-enylglycerol (C3)

Obtained from **C21**, as light yellow oil; 62.4 mg (61.3%); TLC: *R*_f _= 0.75 (CH_2_Cl_2_:MeOH, 95:5 v/v); ^1^H NMR (in CDCl_3_): δ in ppm 0.86 (3H, t, *J *= 7.0 Hz), 0.94 (3H, t, *J *= 7.5 Hz), 1.25-1.29 (20H, m), 1.51-1.55 (4H, m), 1.99-2.07 (6H, m), 2.15 (1H, br s), 2.40-2.45 (4H, m), 2.77-2.83 (10H, m), 3.40-3.45 (2H, m), 3.53-3.64 (2H, m), 3.77-3.78 (2H, m), 4.98 (1H, quintet, *J *= 5.0 Hz), 5.33-5.36 (14H, m); ^13^C NMR (in CDCl_3_): δ in ppm 14.3, 14.4, 20.7, 22.7, 22.8, 25.7, 25.8 (2), 26.2, 27.4, 29.4, 29.5, 29.6 (2), 29.7, 29.9 (3), 32.1, 34.1, 64.3, 70.8, 72.0, 72.5, 127.2, 127.9, 128.0, 128.2, 128.3 (2), 128.4 (2), 128.5, 128.7, 129.7, 129.9, 130.1, 132.2, 173.1; FT-IR (cm^-1^) 3385 (br), 2917, 2852, 1722; HRAPCI-MS *m/z*: measured 653.5509 ([M + H]^+^, calcd. 653.5509 for C_43_H_73_O_4_); APCI-MS/MS *m/z*: 653 ([M + H]^+ ^70%), 635 (30%), 385 (35%), 329 (15%), 311 (100%), 293 (65%), 269 (20%), 97 (30%).

### Cell lines

**Figure 2:**
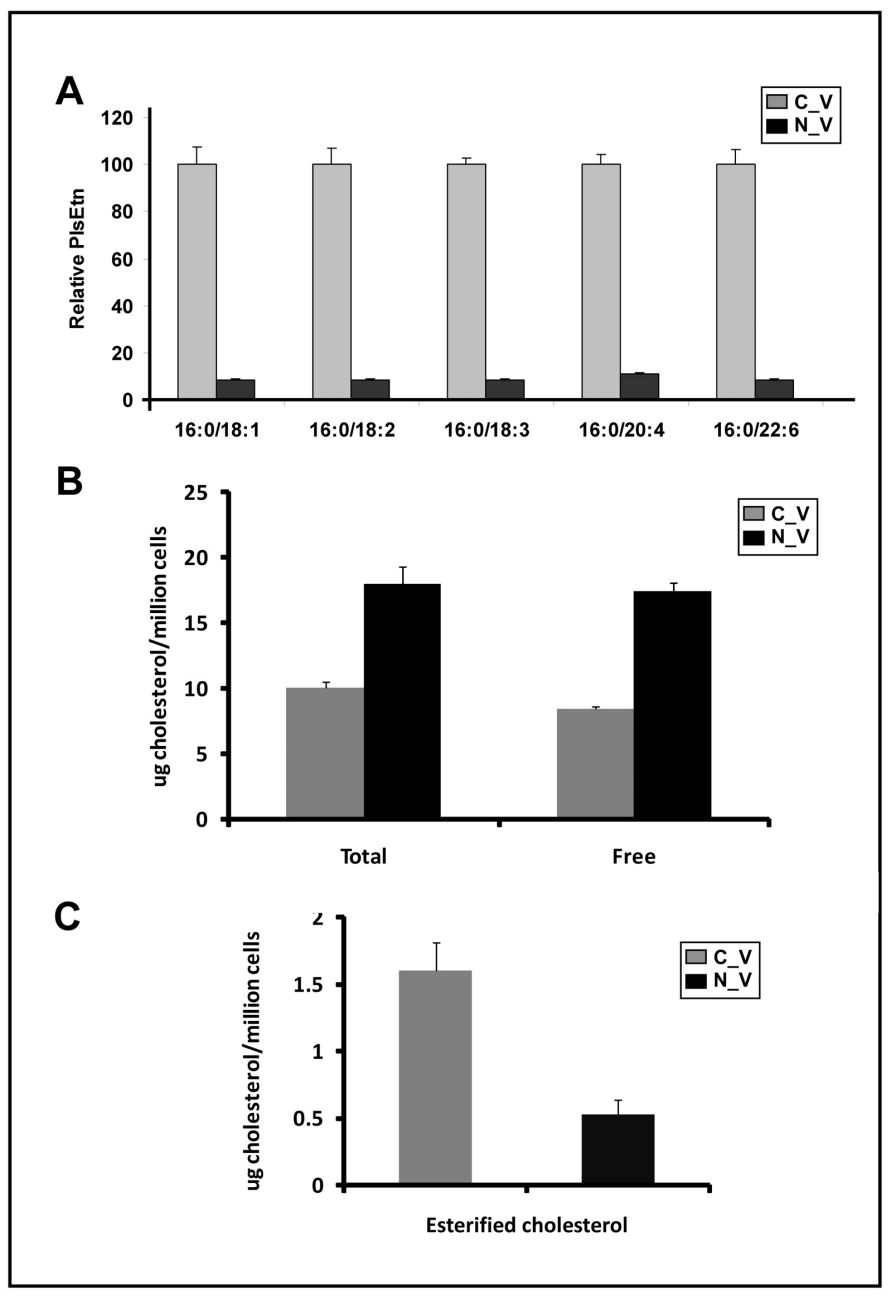
**Relative ethanolamine plasmalogens in DHAPAT-deficient cells (A)**. Plasmalogen content of CHO (C_V) and NRel-4 (N_V) cell lines. Values are an average of three independent experiments; error bars represent standard deviation. All transitions measured in NRel-4 cells were significantly different from control cells (p < 0.05). Cholesterol profile of DHAPAT-deficient cells (B). Values are an average of independent experiments; error bars represent standard deviation. Total, free and esterified cholesterol of N_V is significantly different from C_V ( p < 0.05).

HEK 293 cells were purchased from ATCC, and cultured in DMEM, 10% FBS at 37°C, 5% CO_2_. CHO and NRel-4 cells were a kind gift from Dr. R.A. Zoeller (Boston University) and were cultured in F-12 medium, 10% FBS at 37°C, 5% CO_2_. NRel-4 cells are deficient in peroxisomal dihydroxyacetonephosphate acyltransferase (DHAPAT; EC 2.3.1.42; Figure 2).

### Q-TRAP analysis

The plasmalogen-deficient CHO cell line (NRel-4) was used to assay the efficacy of test compounds **C1**-**10 **in plasmalogen restoration. CHO (wild type) or NRel-4 (plasmalogen deficient) cells were seeded in DMEM/F-12 medium (10% FBS) on a 10 cm dish the day before the experiment. The following day, the media was replaced with fresh media containing the test compound or the solvent ethanol (0.1% V/V) as a control [46]. Cells were cultured for 72 hours at 37°C, 5% CO_2_, after which they were harvested using Versene/TrypLE express (Gibco Life Technology, Rockville, MD). The cell pellet was washed with phosphate buffered saline (PBS, pH 7.4), and the phospholipids were extracted and analyzed using a linear ion-trap mass spectrometer coupled to a LC system as described [31].

### Cholesterol Assay

Human embryonic kidney 293 (HEK293) cells and CHO/NRel-4 cells were seeded the day before the treatment. The following day, the cells were treated with the test compounds **C1**-**10 **or with ethanol as the control. Concentrations used for pravastatin, clofibrate, and troglitazone treatments were as described [47-49]. Cells were harvested after 48 hours using Versene: TryPLe express cocktail, washed with PBS. Lipids were extracted with chloroform containing 1%Triton X-100. The organic fraction was recovered and dried under a stream of nitrogen. The dried lipids were resuspended in cholesterol reaction buffer (Biovision, Mountain View, CA), and the total, free and esterified fractions of cholesterol were quantified using the cholesterol quantification kit (Biovision, Mountain View, CA) as per the manufacturer's recommendations. Cholesterol was reported as μg/million cells.

### Immunoblotting and Immunoprecipitation

HEK293 cells were treated as described in the amyloid assay. The cell pellet was washed in PBS and lysed in RIPA buffer containing a protease inhibitor cocktail (Sigma, St. Louis, MI). Protein in the cell lysate was quantified using the Bio-Rad Protein Assay (Bio-Rad, Hercules, CA). The following antibodies were used for western analyses: SOAT1 (Santa Cruz Biotechnology Inc., CA) and β-actin (Sigma, St. Louis, MI). Band intensities were calculated using ImageJ (National Institutes of Health).

### Statistical analysis

Statistical Analysis of the data was performed using Microsoft Office Excel 2007 and JMP version 8. Multiple comparison Dunnett's tests were applied to analyze the differences between the treatments and the control.

## Results

### The Effect of Plasmalogen Deficiency on Membrane Cholesterol Composition

Membrane plasmalogen levels of NRel-4 cells, lacking dihydroxyacetone phosphate acyl transferase (DHAPAT), an obligate enzyme in the plasmalogen biosynthesis pathway [50,51], were less than 10% of wild-type CHO cells ( Table 2 and Figure 2A) as assessed through the relative quantification of five common palmityl ether ethanolamine plasmalogens (PlsEtn). The cell membranes of NRel-4 cells also contained nearly 2-fold more free cholesterol (Figure 2B) and 4-fold less esterified cholesterol than CHO cells (Figure 2C).

**Table 2:** Ethanolamine plasmalogen distribution in cell lines

Absolute Signal	CHO	NRel-4	HEK293
**PlsEtn 16:0/18:1**	4.935 (11.6)	0.385 (9.2)	5.860 (13.7)

**PlsEtn 16:0/18:2**	4.650 (10.9)	0.405 (9.7)	0.944 (2.2)

**PlsEtn 16:0/18:3**	0.069 (0.2)	0.008 (0.2)	0.127 (0.3)

**PlsEtn 16:0/20:4**	5.900 (13.8)	0.997 (23.9)	9.053 (21.1)

**PlsEtn 16:0/22:6**	0.442 (1.0)	0.093 (2.2)	2.737 (6.4)

			

**PlsEtn 18:0/18:1**	3.580 (8.4)	0.273 (6.5)	2.840 (6.6)

**PlsEtn 18:0/18:2**	4.155 (9.7)	0.312 (7.5)	0.556 (1.3)

**PlsEtn 18:0/18:3**	0.059 (0.1)	0.004 (0.1)	0.072 (0.2)

**PlsEtn 18:0/20:4**	6.300 (14.8)	0.660 (15.8)	5.780 (13.5)

**PlsEtn 18:0/22:6**	0.491 (1.1)	0.104 (2.5)	1.787 (4.2)

			

**PlsEtn 18:1/18:1**	3.805 (8.9)	0.213 (5.1)	4.227 (9.9)

**PlsEtn 18:1/18:2**	3.390 (7.9)	0.185 (4.4)	0.623 (1.5)

**PlsEtn 18:1/18:3**	0.065 (0.2)	0.003 (0.1)	0.078 (0.2)

**PlsEtn 18:1/20:4**	4.390 (10.3)	0.485 (11.6)	6.170 (14.4)

**PlsEtn 18:1/22:6**	0.431 (1.0)	0.048 (1.1)	2.0167 (4.7)

			

**Total PlsEtn Sum**	42.660 (100)	4.173 (100)	42.870 (100)

Absolute signal intensities of the various plasmalogen species measured in CHO, NRel-4 cells, and HEK293 cell lines. Values in brackets are the percentage of total PlsEtn sum.

### The Effect of Plasmalogen Precursor sn-1 and sn-2 Substituents on Plasmalogen Composition in CHO and NRel-4 Cells

**Figure 3:**
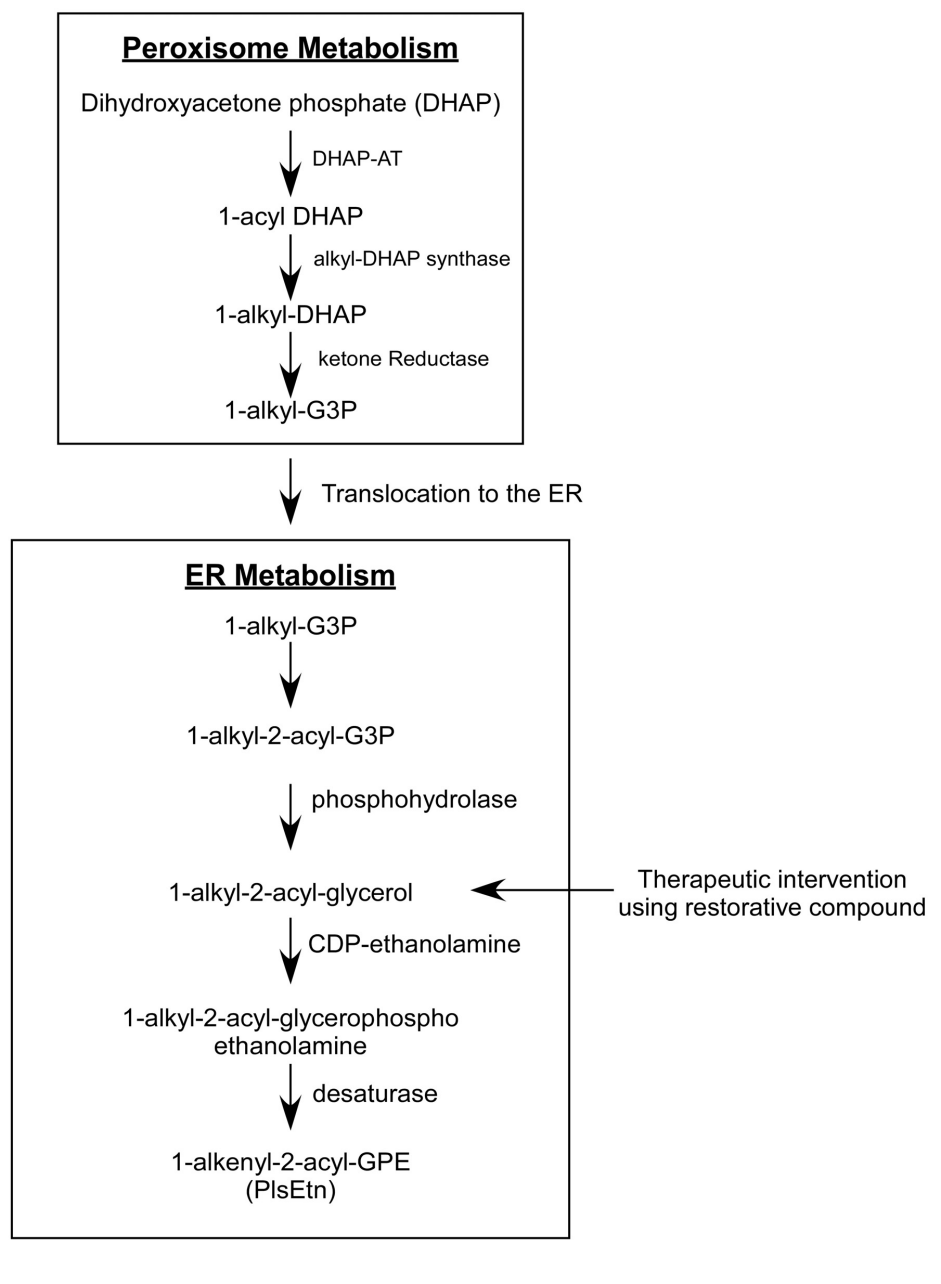
**Plasmalogen biosynthetic pathway showing therapeutic intervention**.

**Figure 4:**
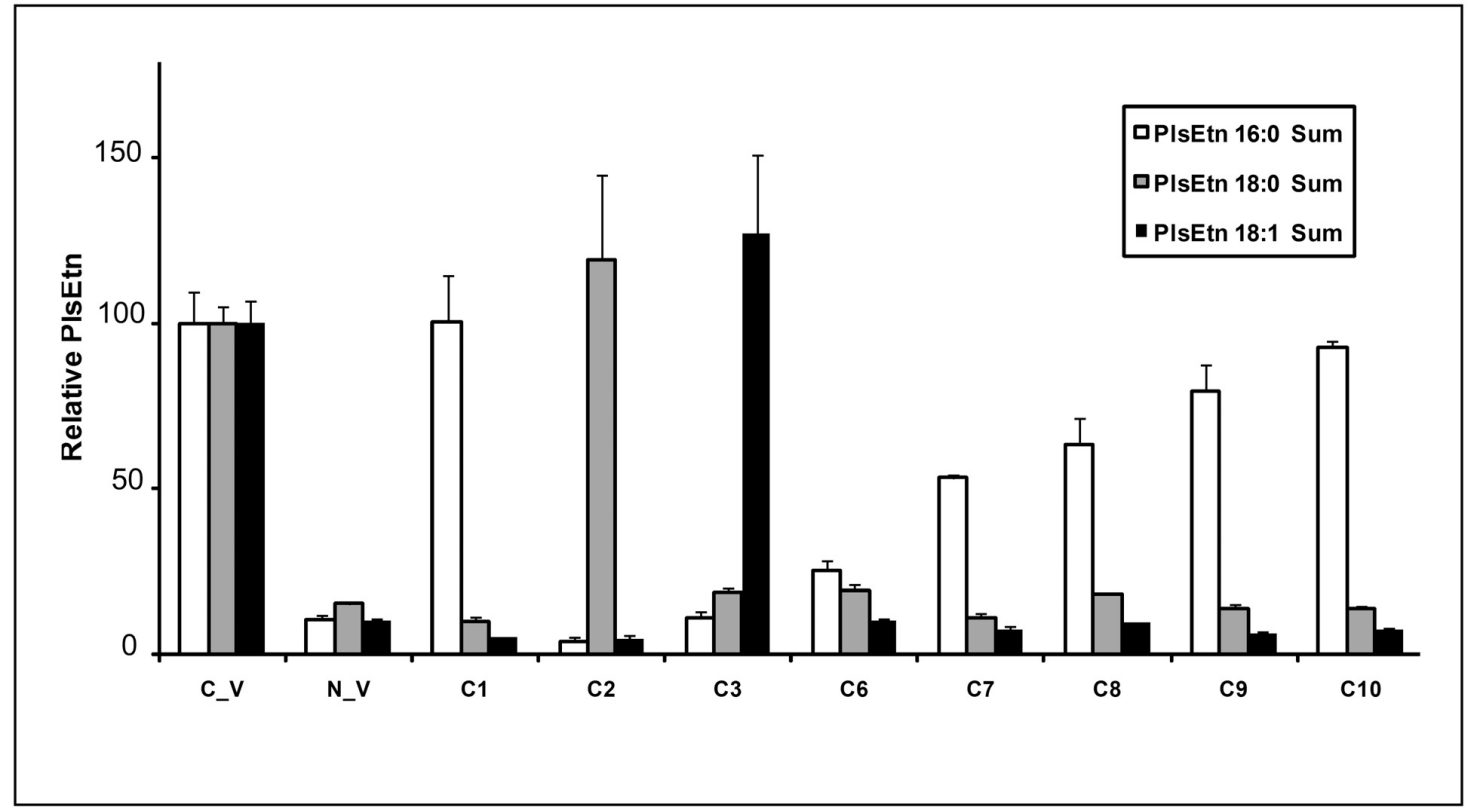
**Side chain-specific restoration of PlsEtn in NRel-4 cells**. Effect of **C1**, **C2**, **C3, C6-10 **treatment of NRel-4 (N_V) cells on sn-1 specific PlsEtn pools.

**Figure 5:**
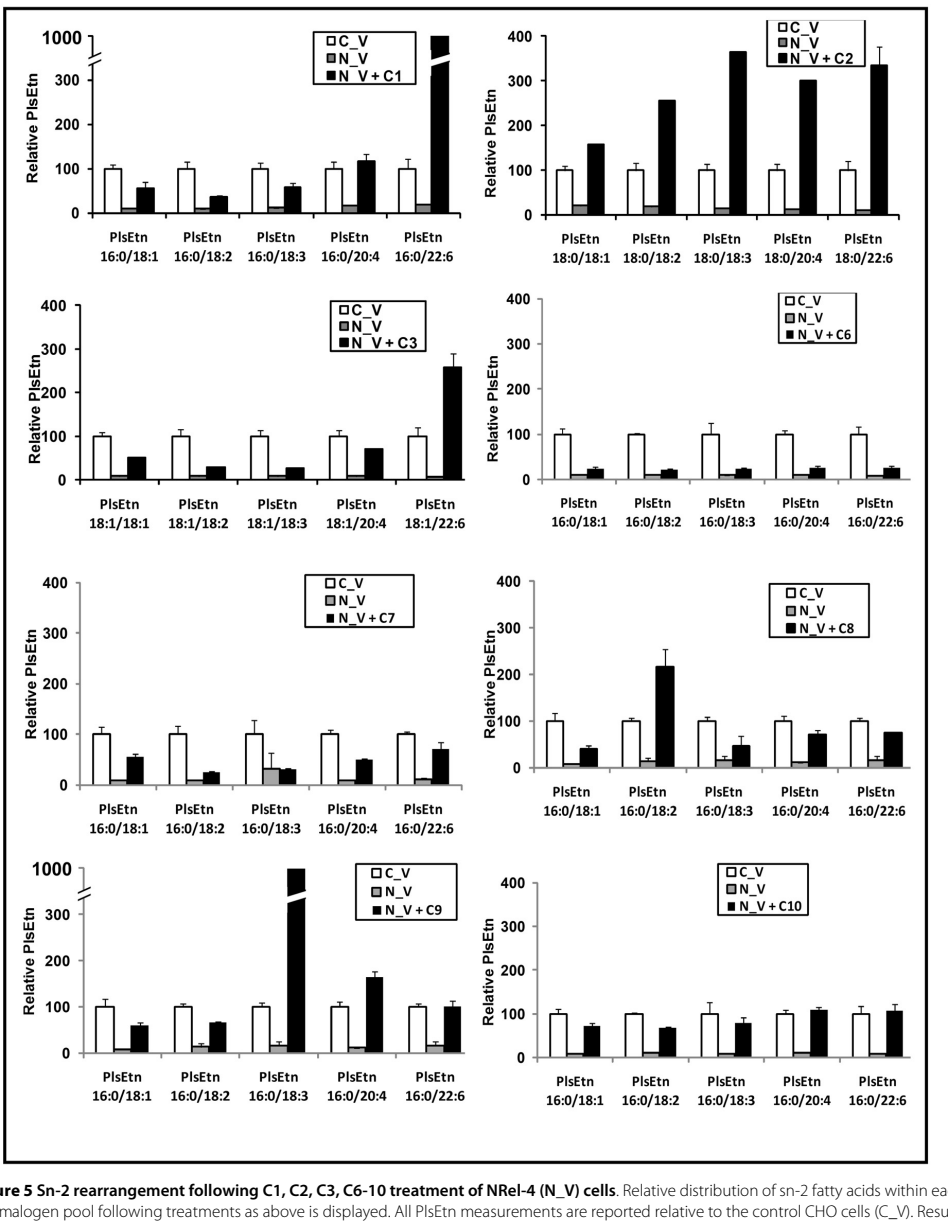
**Sn-2 rearrangement following **C1**, **C2**, **C3, C6-10 **treatment of NRel-4 (N_V) cells**. Relative distribution of sn-2 fatty acids within each plasmalogen pool following treatments as above is displayed. All PlsEtn measurements are reported relative to the control CHO cells (C_V). Results are an average of three independent experiments. Error bars represent standard deviation.

**Figure 6:**
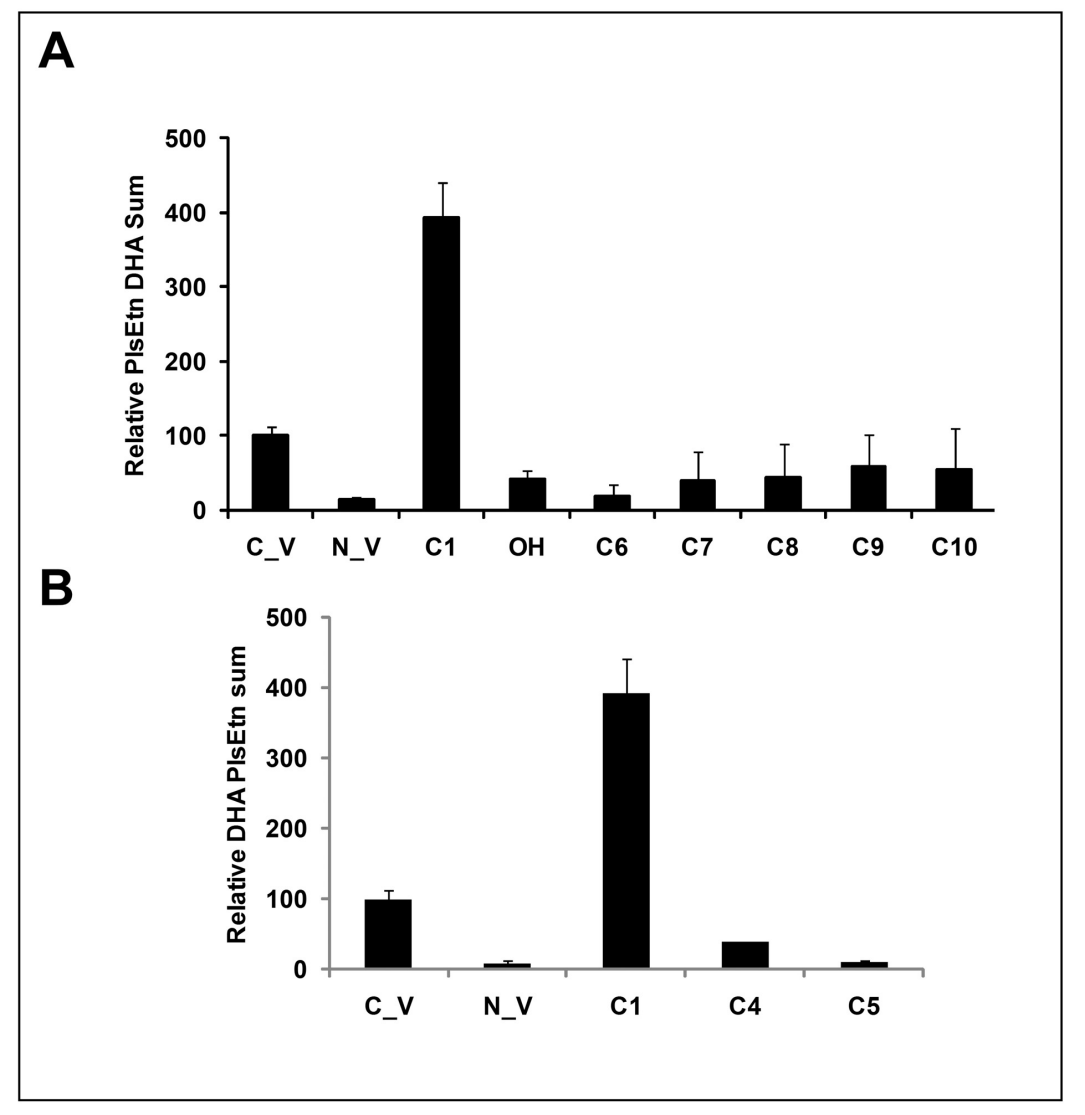
**Comparison of *sn*-2 fatty acid substitution (A) and *sn*-1bond type (B) on total DHA PlsEtn levels in NRel-4 cells**. NRel-4 cells were treated with ethanol solvent (N_V), or with test compounds at 20 μM concentration. Compounds **C1**, **C6-10 **contain palmityl ether at *sn*-1 and different fatty acid moieties at *sn*-2 position; DHA (**C1**), oleic acid (**C7**), linoleic acid (**C8**), linolenic acid (**C9**), and arachidonic acid (**C10**), -OH refers to a free hydroxyl group at *sn*-2 position. Compounds **C4 **and **C5 **have acyl linkages at sn-1 and sn-2 positions. Total DHA containing ethanolamine plasmalogens were quantified, and expressed relative to the amount observed in wild-type CHO cells (C_V). Values were an average of three independent experiments. Error bars represent standard deviation.

**Figure 7:**
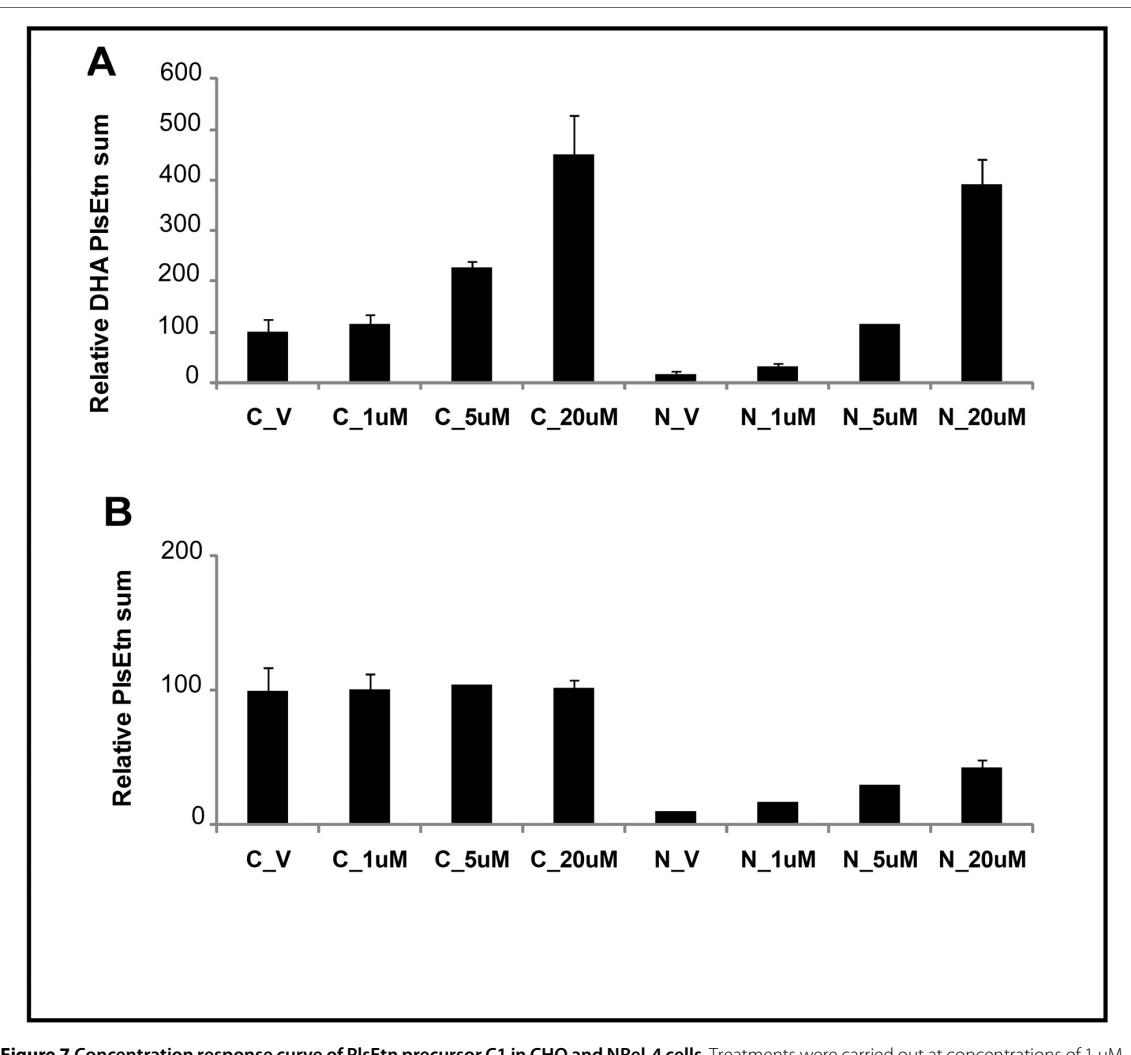
**Concentration response curve of PlsEtn precursor **C1 **in CHO and NRel-4 cells**. Treatments were carried out at concentrations of 1 μM, 5 μM, and 20 μM of **C1**. (A): Relative restoration/augmentation of DHA PlsEtn; (B): Relative restoration/augmentation of total PlsEtn. Values were normalized to control CHO cells (C_V). Results are an average of three independent experiments. Error bars indicate standard deviation.

Using wild CHO and NRel-4 cells, side chain-specific PlsEtn and phosphatidylethanolamine (PtdEtn) precursors (Table 1) were evaluated for their abilities to augment cellular plasmalogen levels in control (CHO) and PlsEtn-deficient (NRel-4) cells. These alkylacylglyceryl ethers bypass the requirement for peroxisomes in the synthesis of plasmalogens (Figure 3). The following observations were made:

1. Maintaining the free alcohol at *sn*-3 and DHA at *sn*-2, PlsEtn precursors **C1**-**3 **with differing long chain ether substitutions at *sn*-1 revealed that these precursor compounds either partially or fully restored all ethanolamine plasmalogens with the same *sn*-1 alkyl ether but had no effect on PlsEtn with different *sn*-1 compositions (Figure 4). For example, treatment with a palmityl PlsEtn precursor (**C1**) restored the downstream pool of 16:0 ethanolamine plasmalogens with no effect on the 18:0 and 18:1 PlsEtn pools. Such side chain-specific restoration indicates that no rearrangement of the *sn*-1 moiety (*O*-alkyl linkage) occurs, while the *sn*-2 moiety is able to undergo deacylation and subsequent reacylation with other fatty acid residues.

2. Similarly, compounds **C6-C10 **(16:0 at *sn*-1 but differing at the *sn*-2 substituent) significantly elevate the 16:0 pool, with no effect on the 18:0 and 18:1 pools of PlsEtn (Figure 4).

3. Distribution of PlsEtn within a pool (16:0, 18:0 or 18:1) depends on the fatty acid at *sn*-1 position. **C1 **and **C3 **showed maximum restoration of the PlsEtn directly downstream in the pathway (16:0/22:6 PlsEtn and 18:1/22:6 PlsEtn respectively). **C2 **on the other hand significantly augments all PlsEtns in the 18:0 pool (Figure 5).

4. Comparison of compounds **C1**, **C6**-**10**, revealed that whereas DHA containing precursors can partially or fully restore all other *sn*-2 PlsEtn, non-DHA containing precursors cannot completely restore DHA-PlsEtn (Figure 6A).

5. DHA-PtdEtn precursors (**C4 **and **C5**) cannot restore DHA-PlsEtn deficiencies (Figure 6B).

6. PlsEtn precursors with DHA at *sn*-2 concentration-dependently increase DHA-PlsEtn in both DHAPAT deficient cells (NRel-4) and wild-type cells (CHO) (Figure 7A). However, with respect to total plasmalogen content, only the deficient cell line showed an increase; no augmentation in total plasmalogen content was observed in wild-type CHO cells (Figure 7B).

### The Effect of Plasmalogen Precursor Structure on Membrane Cholesterol Composition

**Figure 8:**
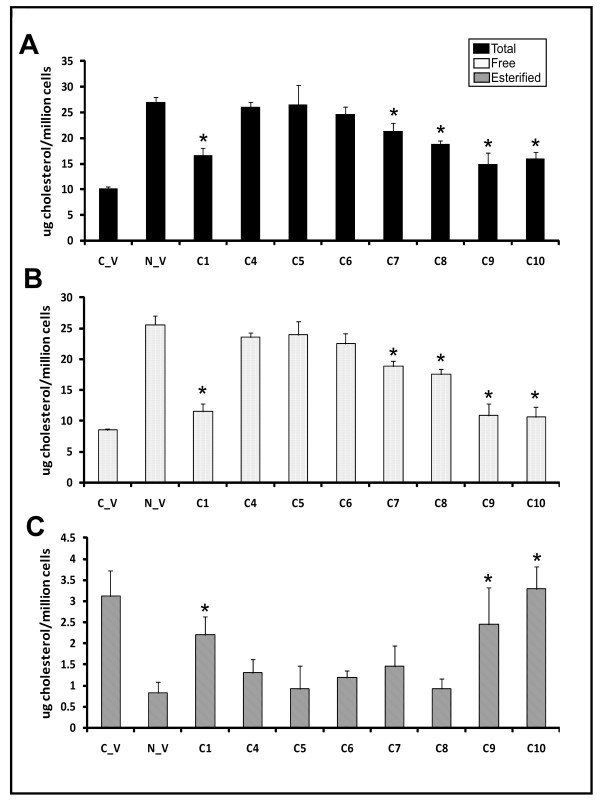
**Cholesterol profile of CHO/NRel-4 cells**. Cholesterol profile of NRel-4 cells following 48 hour treatments with PlsEtn precursors (C1, C6-10) and PtdEtn precursors (C4, C5) compared to control CHO cells. A: total cholesterol; B: free cholesterol; C: esterified cholesterol. Cholesterol is reported as μg per million cells. Results are an average of three independent experiments. Asterisk represents values that are significantly different from those observed in DHAPAT-deficient NRel-4 cells (p < 0.05).

As demonstrated above, plasmalogen deficient cells have higher content of free cholesterol and lower amounts of esterified cholesterol in their cell membranes. To determine whether this effect was due to a general decrease in membrane PlsEtn composition or to decreased levels of specific PlsEtn, membrane PlsEtn levels in PlsEtn depleted cells (NRel-4) were selectively restored as described above and the corresponding effect on membrane cholesterol composition ascertained. The key observations were:

1. PtdEtn precursors (**C4 **and **C5**) had no effect, while PlsEtn precursors with < 3 unsaturations (**C7 **and **C8**) had a mild effect on membrane cholesterol composition (Figure 8).

2. PlsEtn precursors with 3 or more unsaturations (**C1**, **C9 **and **C10**) had a more profound effect on reducing free cholesterol (Figure 8B) and increasing esterified cholesterol (Figure 8C).

**Figure 9:**
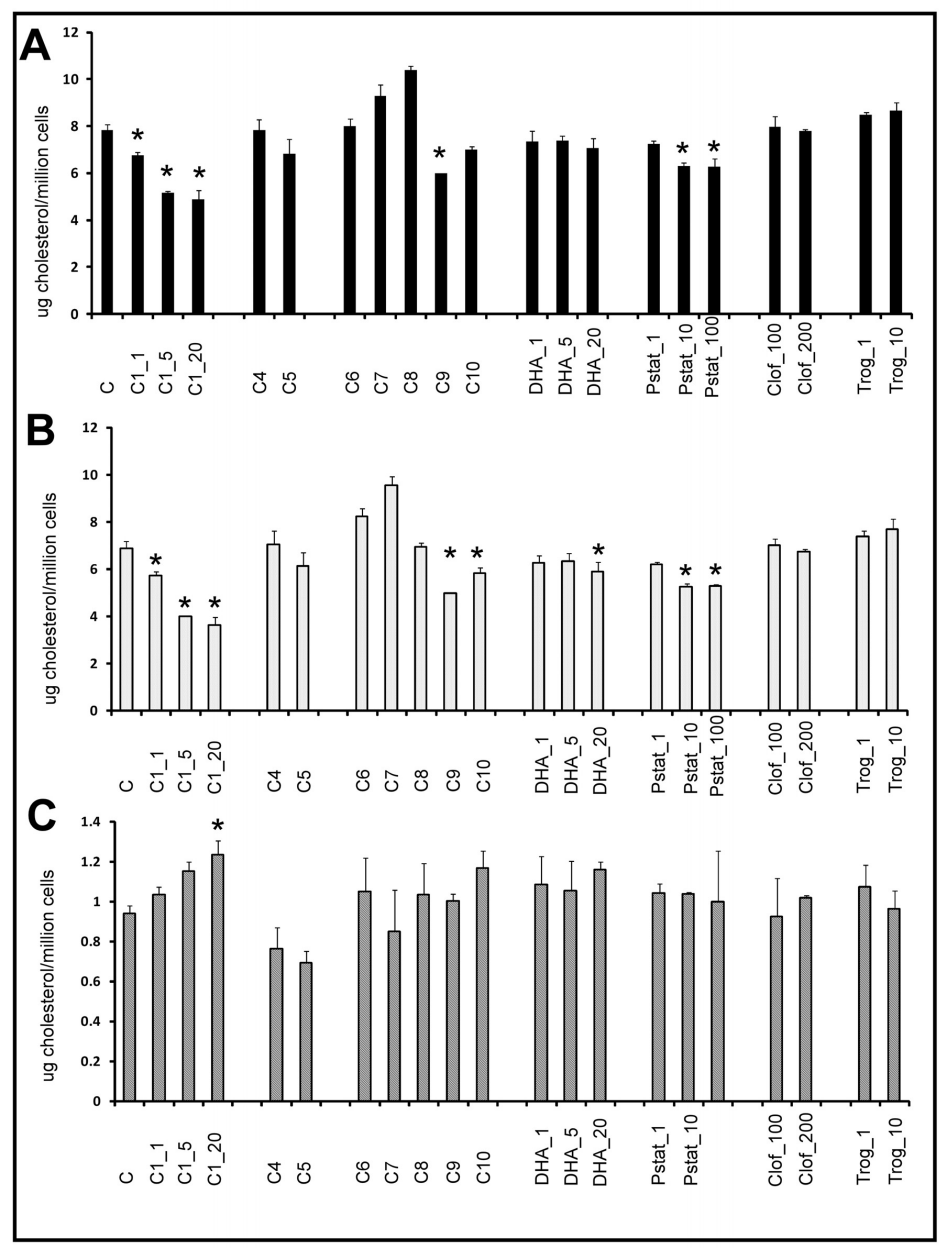
**Cholesterol profile of HEK293 cells treated for 48 hr with plasmalogen precursors**. Cholesterol (total, free and esterified) content is reported as μg/million cells, and is an average of two independent experiments. Error bars indicate standard deviation. Asterisk indicates significantly lower total or free cholesterol, or significantly higher esterified cholesterol compared with control.

The effect of plasmalogen precursors and other compounds on membrane cholesterol composition was further studied in PlsEtn normal human HEK293 cells (Figure 9). The key observations were:

1. PlsEtn precursor **C1 **exhibited a concentration-dependent decrease in free cholesterol (47% at 20 μM) (Figure 9B) and a reciprocal increase in the esterified fraction of cholesterol (31% at 20 μM) (Figure 9C)

2. PtdEtn precursors (**C4 and C5**) had no effect on free cholesterol and resulted in slight decreases in esterified cholesterol.

3. PlsEtn precursors with *sn*-2 substituents containing < 3 unsaturations (**C6, C7 and C8**) either elevated or had no effect on free cholesterol.

4. PlsEtn precursors with *sn*-2 substituents containing 3 or more unsaturations either reduced free cholesterol (**C1 **and **C9**) and/or increased esterified cholesterol (**C1 **and **C10**).

5. Free DHA had a slight impact on free cholesterol (14% reduction) compared to control, while it exhibited a 24% increase in the esterified cholesterol fraction at the 20 μM concentration.

6. Pravastatin treatments were most potent in reducing free cholesterol at 10 μM concentration (24% reduction compared to control, p = 0.01), while the 100 μM concentration did not result in a further reduction of free cholesterol. The changes observed in the esterified cholesterol were not significant.

7. Treatments with PPARα (clofibrate; 100 and 200 μM) and PPARγ (troglitazone; 1 and 10 μM) agonists had no impact on the cholesterol profile of HEK293 cells at the concentrations tested.

### Effect of PUFA-PlsEtn enhancement and HMG-CoA inhibition on cellular SOAT1 levels

**Figure 10:**
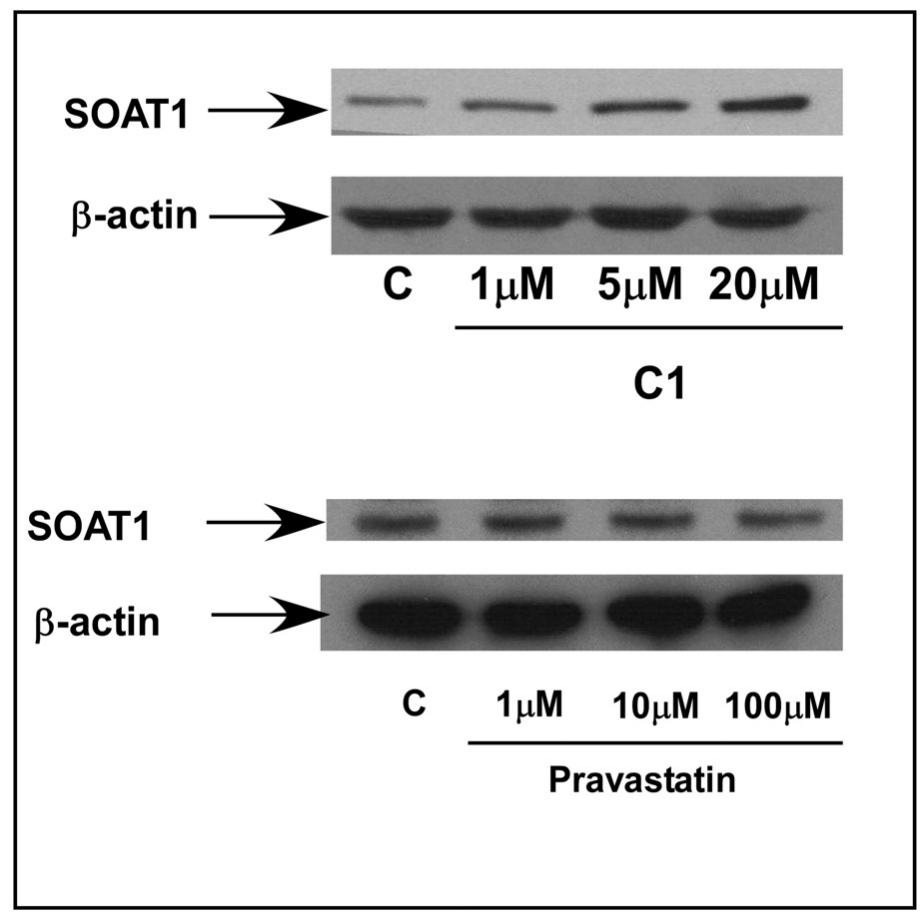
**Immunoblots showing SOAT1 protein levels in wild-type HEK293 cells treated with a concentration range of C1 and pravastatin**. β-actin was used as a loading control.

The effects of the potent cholesterol esterification enhancing/total cholesterol lowering PlsEtn precursor, **C1**,and of the potent HMG-CoA reductase inhibiting/total cholesterol lowering statin, pravastatin, on the basal levels of cholesterol processing enzyme SOAT1 was determined. The maximum cholesterol-lowering concentration of **C1**, resulted in a 50% elevation of SOAT1 levels (calculated using ImageJ software), whereas the maximum cholesterol-lowering concentration of pravastatin had no effect on SOAT1 levels (Figure 10).

## Discussion

Plasmalogens are major structural and functional lipids of the cell. The discovery of this class of molecules was made originally in myelin by Feulgen and Voit in 1924 [52], but the accurate structure of plasmalogens was deduced only several years later [53,54]. The biological function of plasmalogens, and their implication in diseases remained elusive for a number of years until a recent spike in interest in these lipids. In this report we discuss the interplay between plasmalogens and cholesterol, and investigate a plasmalogen restoration approach *in vitr*o.

The plasma membrane is the major storage location of free cholesterol in that 80 to 95% of total cellular cholesterol is found there, dependent upon cell type [55-58]. Excess cholesterol is transported out of peripheral cells via HDL proteins following esterification in the membrane and back to the liver via a process called reverse transport [59,60]. Within the cell, cholesterol is transported from the plasma membrane to other cellular compartments via LDL after esterification within the membrane [61,62].

PlsEtn deficient cells have been previously shown to have impaired HDL-mediated cholesterol efflux [38] and impaired intracellular LDL-mediated transport [39]. In both of these studies normal functionality was observed by either PlsEtn precursor treatment [38] or re-instatement of the PlsEtn biosynthesis pathway [39]. Our data is consistent with these studies in that plasmalogen deficient cells were observed to have reduced levels of esterified cholesterol and increased levels of free and total cholesterol (Figure 8) in the membrane. We have expanded upon these studies by investigating in greater detail the effect of membrane PlsEtn speciation on membrane cholesterol esterification. By using a PlsEtn deficient cell model (NRel-4) we selectively restored different PlsEtn species by treating the cells with different 1-alkyl- 2-acyl glycerols (PlsEtn precursors). A comparison between precursors **C1**, **C2**, and **C3 **(which differ only in the *sn*-1 fatty acid) revealed that the *sn*-1 substituent affected rearrangement at *sn*-2 position (Figure 5), and hence the downstream restoration of plasmalogens. This effect could be explained on the basis of stability of the compounds. The DHA moiety in **C2 **is possibly more labile (compared with **C1 **and **C3**) due to the steric hindrance caused by 18:0 fatty acid at sn-1 position.

These structure activity relationships revealed that changes in membrane PUFA-PlsEtn levels are principally responsible for the observed cholesterol effect (Figure 8) and that restoration of membrane PUFA-PlsEtn levels restores cholesterol homeostasis. The effect of membrane PlsEtn modification using 1-alkyl-2-acyl glycerol PlsEtn precursors on membrane cholesterol homeostasis was further investigated using a human cell line (HEK293) with normal PlsEtn biosynthetic machinery. PlsEtn precursor **C1 **concentration-dependently increased membrane esterified cholesterol (Figure 9C) and decreased free and total cholesterol (Figure 9A, B). Additionally, PUFA-PlsEtn precursors were observed to be approximately twice as effective as statins at lowering cholesterol levels. Treatment of the cells with DHA showed a slight, yet significant reduction in free cholesterol in agreement with the literature [63]. These results, in combination with the detailed study of Munn and coworkers [39] strongly indicate that PUFA-PlsEtn precursors reduce membrane cholesterol levels via increased membrane cholesterol esterification and transport. While it is true that treatment of cells with PUFA-PlsEtn results in greater esterification of cholesterol, it is not expected to result in conditions similar to those in cholesterol ester storage diseases, Cholesterol storage disease is caused by lesions in the gene encoding lysosomal acid lipase. In conditions where the lysosomal enzyme is intact, it is expected that the cholesterol esters would be efficiently packed into high density lipoprotein complexes to form HDL-cholesterol for reverse cholesterol transport.

The observed increase in cholesterol esterification is suggested to be due to elevated SOAT1, an enzyme expressed in liver cells and macrophages which is involved in cholesterol homeostasis (Figure 10). These observations explain the increase in esterified cholesterol and an elevated rate of HDL-mediated cholesterol efflux reported by others [38,39]. These effects could not be reproduced by either PPAR agonists or by HMG-CoA reductase antagonists indicating that membrane PUFA-PlsEtn enhancement is a novel mechanism for lowering membrane cholesterol levels.

It is prudent to note that ACAT inhibition was thought to be a promising pharmaceutical target for controlling hypercholesterolemia. Several ACAT inhibitors entered clinical trials, only to emerge with disappointing results. Avasimibe and pactimibe treatment did not hamper the progression of coronary atherosclerosis [64,65]. On the contrary, in both trials the ACAT inhibitors resulted in a significant elevation in LDL cholesterol over the placebo arm, prompting an early termination of the trials. Additionally, in pactimibe trials, the treatment groups showed a significant increase in atheroma volume in the coronary artery [64], and significant increase in carotid intima-media thickness compared to the placebo group [66]. These data question the strategy of ACAT inhibition in treating hypercholesterolemia. Our data on the other hand suggests that an increase in SOAT1 expression is key to the formation of cholesterol esters (and reduction in free cholesterol) prior to HDL mediated cellular cholesterol efflux.

In summary, using a series of 1-alkyl-2-acylglycerols, we showed that membrane PlsEtn levels can be selectively restored in a PlsEtn deficient system and selectively augmented in PlsEtn normal cells in a concentration-dependent manner. Accordingly, these results represent the first report of selective plasmalogen enhancement in normal cells. The structure activity relationship study suggests that selective PUFA-PlsEtn enhancement is capable of beneficially favoring cholesterol esterification, an obligate step prior to efflux from the cell. This translates to a net reduction in the fraction of free cholesterol in cells. Plasmalogen restoration/enhancement therefore offers a novel mechanism of cholesterol reduction *in vitro*.

## Competing interests

PLW is CEO of and owns stock in Phreedom Pharma.

DBG is CEO of and owns stock in Phenomenome Discoveries Inc.

## Authors' contributions

RM designed and conducted experiments, prepared the manuscript. PWKA and DJ synthesized compounds used in the study. HM carried out experiments. KKS carried out statistical analyses. MAK participated in design of experiments. SR, PLW and DBG participated in design and manuscript preparation. All authors read and approved the manuscript.
